# Bridging the gap: implementation of an online induction course to support students’ transition into first year medicine

**DOI:** 10.15694/mep.2020.000193.2

**Published:** 2021-03-17

**Authors:** Kirsty McIntyre

**Affiliations:** 1 University of Glasgow

**Keywords:** Transition, induction, online, digital skills, professionalism, medical students, undergraduate medicine, medical education.

## Abstract

This article was migrated. The article was marked as recommended.

Introduction

Students transitioning into medical school encounter a series of transitions on their journey towards graduation. In the context of undergraduate medicine, students must develop new skills common to all incoming undergraduates (e.g., academic writing, digital skills), as well as subject-specific knowledge, e.g., regarding professionalism or anatomical dissection governance. Whilst the University of Glasgow School of Medicine provides on-campus induction activities, e.g., to prepare students to participate in problem-based learning, other induction material has traditionally been delivered in a didactic manner.

Methods

We developed a five-unit online induction course using existing resources available under creative commons licensing. The course used an interactive approach to deliver teaching and signpost key resources related to digital and academic skills and professionalism. The course was released to incoming students ( *n* = 316) via the institutional public Moodle site prior to their arrival at Glasgow. Student engagement and perception was assessed using quizzes, an online anonymous survey ( *n* = 133) and focus group ( *n* = 2).

Results

Student engagement with the induction course was high: 95% (301/316) of students accessed the course, and 89% (280/316) of students completed the course by achieving 100% in all five end-of-unit quizzes. Students placed particular value on content relating to professional expectations and highlighted that inclusion of current students’ testimonies would improve the course. However, the requirement to engage with sections relating to fundamental digital skills was sometimes a barrier to engagement.

Discussion

Overall, the online induction course benefitted students by helping them understand institutional expectations and creating an opportunity to identify their digital skills needs for them to succeed in their studies. These insights will be of key importance in supporting transition as we prepare for remote induction of our students in September 2020 and should be applicable to others interested in adapting and implementing this induction course in their own settings.

## Introduction

Entry to first year medicine (MBChB) is a major transition for students on their journey to becoming doctors. In common with all incoming undergraduates at the University of Glasgow, medical students must develop their digital literacy, academic writing and self-directed learning skills (University of Glasgow, no date). Successful integration into medical school also requires navigating new concepts such as professionalism and anatomical dissection governance.

Induction activities to support student transition into higher education are key to ensuring student retention and success. The transition period is affected by an individual’s capability to navigate change, with many students experiencing ‘academic culture shock’ ( Quinn *et al.*, 2005). The majority of withdrawals in the general student population (approximately two-thirds) happen within the first year of university ( Yorke, 2010). In the UK, 1-14% of medical students leave before graduation, most during the first year ( Simpson and Budd, 1996; Yates, 2012; Higher Education Statistics Agency, 2019). For those who do continue, evidence from a longitudinal study of medical students indicates that students who are the most distressed at the beginning of their medical education remain so throughout their programme of study ( Niemi and Vainiomäki, 2006). Specialised support and guidance at this vital time is thus essential to help our students adapt and flourish in their new environment. Many studies have investigated how transition affects those from widening participation backgrounds as undergraduate populations diversify (e.g. Brosnan *et al.*, 2016; Bassett *et al.*, 2018). However, we must ensure that all students are adequately supported throughout their first year of study: little is known about the preparedness of our cohort as a whole.

In our institution, student transition to problem-based learning (PBL) is well supported during integration week (Freshers’ week), as recommended in the literature ( Moro and McLean, 2017). Didactic lectures have traditionally supported other aspects of student transition into medical education. However, students still frequently contacted staff to ask for the information provided in these sessions, which suggested that this approach was not adequately preparing our students.

This academic year (2019-20) we chose a blended learning approach by “combining face-to-face interactions with online activities” ( Advance HE, 2019). We developed a new five-unit online induction course using existing resources available under creative commons licensing (see Table 1 for course overview) (Bloomsbury Learning Exchange, no date). The course was released to incoming students via the institutional public Moodle (VLE) site prior to their arrival at Glasgow and was supplemented by face-to-face activities during integration week. The online induction course used an interactive approach to deliver teaching, and support students in developing their academic and digital skills. Students were able to navigate the course, which contained short written excerpts and videos, links to additional resources, and a virtual reality tour of the University medical school building, at their own pace. Moreover, in line with previous recommendations ( Harvey, Drew and Smith, 2006) the course introduced students to key members of staff and to the core curriculum. At the end of each unit there was a quiz in which students were required to achieve a score of 100% (unlimited attempts) to complete the course. On completion the students were awarded a digital certificate.

**Table 1:** Overview of the components of the online induction course as launched in September 2019

Unit	Title	Summary of content
1	General technologies	Assistive technology, working with files, Office applications and search engines.
2	Learning technologies	Virtual learning environment (Moodle), lecture capture, assessments and plagiarism.
3	Access, sharing and safety	Accounts, access (e.g., to Wifi) and logins. Communication tools such as email and social media.
4	Getting organised	Note-taking, referencing and digital wellbeing.
5	Medicine at Glasgow	Introduction to key staff, curriculum overview, virtual reality tour of University medical school building. Introduction to professionalism and our expectations of our students.

An existing Digital Skills Awareness course (Bloomsbury Learning Exchange, no date) was the framework for units 1-4. To this, institutional links were added, and the medicine-specific course content developed for unit 5.

Remote induction of students entering higher education in September 2020 is likely due to the ongoing COVID-19 pandemic. Adaptation and refinement of this online induction course will therefore be key in supporting students during this time.

This study aimed to assess student perceptions of the induction course including how, and if, they felt that the course has supported them during their first year of study.

## Methods

Two approaches to data collection were used. Firstly, an online survey was implemented to collect data relating to perceptions of the course from a large proportion of the year group. Secondly, to collect rich qualitative data regarding students’ perceptions of the usefulness of the online induction course in preparing them for first year medicine, a focus group was conducted.

### Institutional setting

This study was conducted at the University of Glasgow School of Medicine during the 2019-20 academic year. Incoming first year medicine (MBChB) students were granted access to an online induction course on the institutional Moodle site before joining on campus for week 0 (Freshers’ week).

### Survey design

All enrolled first year medical students were invited to complete an anonymous questionnaire about their perceptions of the induction course after completion. The online questionnaire was designed by the author and contained nine questions in total (Supplementary File 1). The participant information sheet was the first page of the online survey, which informed students that participation inferred consent.

Survey participants were asked to indicate their previous training (e.g. school leaver, graduate) and, based on this, how prepared they felt entering the medical programme (five-point Likert scale). No personal identifying information (gender, ethnicity) was collected. The main body of the survey contained seven statements to be rated on a five-point Likert scale related to the perceived utility and appropriateness of the induction course. Participants were also asked to rate the helpfulness of components of the induction course (nine items; ‘not at all’, ‘somewhat’ or ‘extremely helpful’). To calculate a Net Promoter Score, a customer satisfaction metric used to measure the likelihood of an individual recommending a product to friends or colleagues ( Customer.guru, 2020), students were asked to indicate how likely it is that they would recommend the induction course to another student starting first year (scale from 0 to 10). Net Promoter Score was calculated as overall percentage (%) of promoters (individuals who selected a score of 9 or 10) minus overall percentage (%) of detractors (those who gave a score between 0-6). Individuals who responded with a score of 7 or 8 were categorised as ‘passives’ and not included in the calculation. Finally, three open text questions prompted students to provide further comment on the course and what influenced their participation.

The questionnaire was advertised via the induction course and the student Moodle site, and was open for participation between 3 ^rd^ September-11 ^th^ October 2019.

### Focus group

Further investigation of student perceptions of the usefulness of the online induction course in preparing them for first year medicine was conducted by focus group. Students were invited to volunteer to participate in the focus group via an announcement on the student Moodle site by a course administrator. The announcement indicated that an independent staff member who was familiar with the medical curriculum would facilitate the focus group. The participant information sheet, which detailed the purpose and outline of the study, was also made available at this point.

Two students volunteered to participate in the focus group, which was held in March 2020. This data collection method was chosen to explore participants’ thoughts and provide more depth and context to the questionnaire data previously collected ( Powell and Single, 1996). The nature of a focus group enables participants to react to and build upon one another’s contributions which is not possible in other methodology e.g. individual interviews ( Stalmeijer, Mcnaughton and Van Mook, 2014).

Students read and signed a participant information form detailing the scope of the study and indicated consent by signing a consent form. The facilitator prompted discussion about the induction course during the session by asking open questions, agreed by prior discussion with the author, related to the research question: What are students’ perceptions of the usefulness of an online induction course for preparing them for first year medicine (MBChB)? Focus group participants were also asked the open text questions from the online questionnaire. Qualitative data was collected in the form of audio recordings of the verbal discussion (lasting approximately 35 minutes) within the focus group setting. The data were transcribed and checked manually to ensure the transcript was an accurate reflection of the recording. Participants and the facilitator were assigned anonymous identifiers (e.g., student (S) 1 or 2, or facilitator (F)) within the transcript; individuals’ names and previous training (e.g., whether or not they were graduates) was not recorded.

The data were coded and analysed using inductive thematic analysis, i.e. themes were identified within the data rather than from a pre-existing coding framework, following the six-step framework by Braun and Clarke (2006). Coding was conducted initially on paper, then using NVivo 12 qualitative data analysis software (QSR International Pty Ltd.) for subsequent rounds of coding and recoding. This iterative process was continued until four distinct themes were identified and named, with multiple distinct codes that classified the data within each theme. Data were not restricted to a code, i.e. items could be coded more than once.

This study was approved by the University of Glasgow College of Medical, Veterinary and Life Sciences Ethics Committee.

## Results/Analysis

There was high engagement with the induction course: 95% (301/316) of enrolled students accessed the course, and 89% of students (280/316) completed the course by scoring 100% in each end-of-unit quiz. Forty-two % of the year group (133/316) participated in the online questionnaire.

When asked about how prepared they felt about entering the undergraduate medical programme based on their previous training, the greatest proportion of school leavers said they felt ‘moderately’ prepared (47.1%, 41/87) whereas the majority of graduate students (53.2%, 13/24) and those who had completed a foundation or pre-med course (50.0%, 7/14) felt ‘very’ prepared ( Table 2).

**Table 2:** How prepared did you feel about entering the medical programme?

	Not at all	Slightly	Moderately	Very	Extremely	Total *n* (total %)
School leavers (%)	5 (5.7)	27 (31.0)	41 (47.1)	11 (12.6)	3 (3.4)	**87 (65.4)**
Graduates (%)	1 (4.2)	1 (4.2)	6 (25.0)	13 (54.2)	3 (12.5)	**24 (18.0)**
Foundation or pre-med course (%)	0 (0.0)	1 (7.2)	4 (28.6)	7 (50.0)	2 (14.3)	**14 (10.5)**
Repeating or returning to year (%)	0 (0.0)	0 (0.0)	1 (50.0)	1 (50.0)	0 (0.0)	**2 (1.5)**
Other (%)	0 (0.0)	1 (16.7)	3 (50.0)	1 (16.7)	1 (16.7)	**6 (4.5)**
**Total *n* (total %)**	**6 (4.5)**	**30 (22.6)**	**55 (41.4)**	**33 (24.8)**	**9 (6.8)**	**133 (100)**

Responses categorised by total participants ( *n*) and by previous training subgroup. Other: gap year ( *n* = 4) mature student ( *n* = 1) transfer student ( *n* = 1).

**Figure 1:**
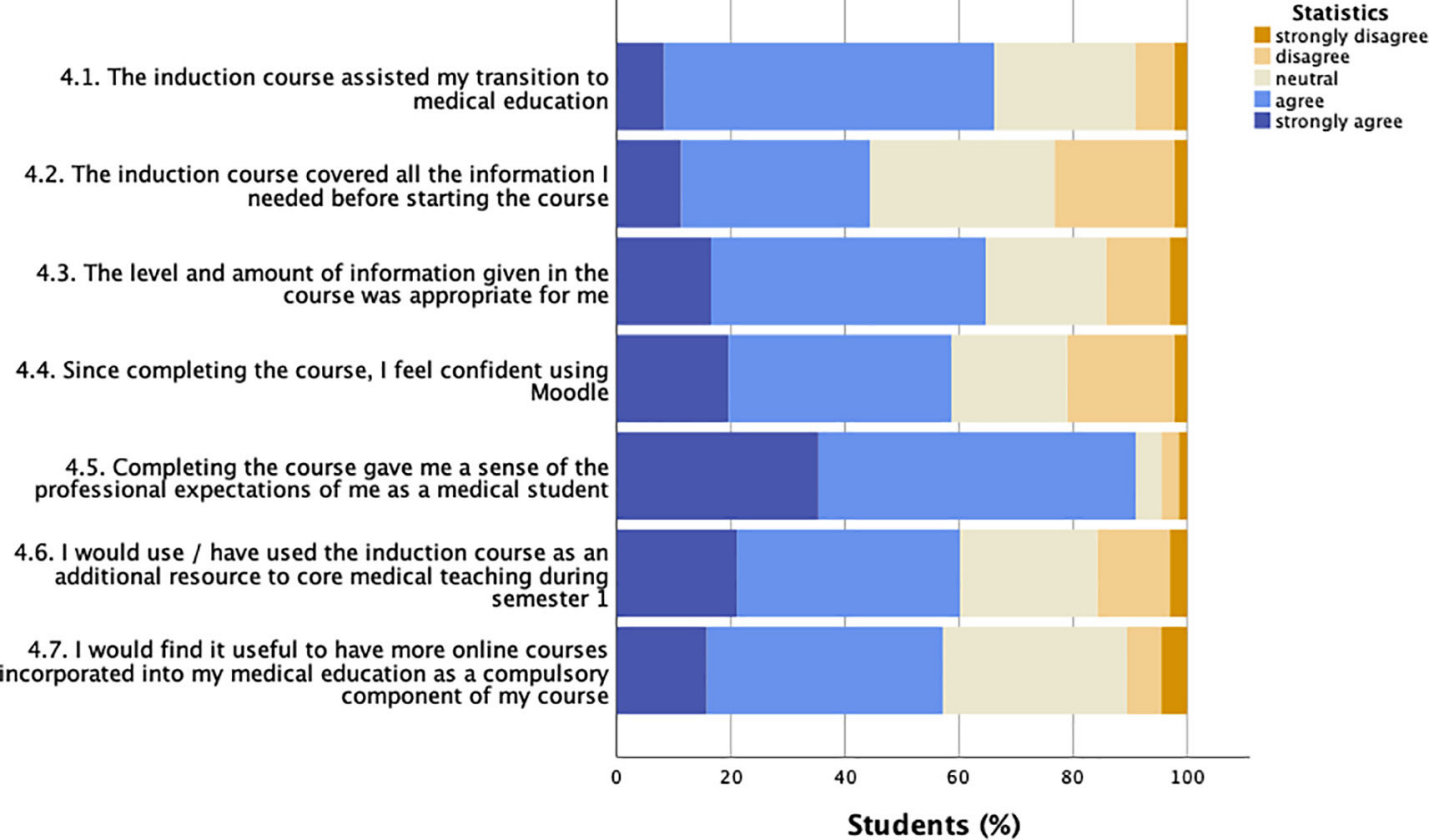
Student responses to induction course statements (Likert scale)

The response to Likert scale statements related to the induction course are illustrated in Figure 1. The majority of students (66.2%, 88/133) agreed or strongly agreed that the induction course assisted their transition to medical education and gave them confidence using the virtual learning environment Moodle (58.6%, 78/133). Information on professionalism was judged as being most valuable by students: 90.9% of participants (121/133) felt that completing the course gave them a sense of the professional expectations of them. In contrast, fewer than half of respondents felt that the induction course covered all the information needed before starting the course (44%, 59/133). Yet, participants rated all components of the course, e.g., short videos, quizzes, links to additional resources, as ‘somewhat’ or ‘extremely helpful’ (mean 94.1%, data not shown).

Questionnaire participants were asked to rate how much they agreed or disagreed with the statements (Y axis labels). Responses are given as % of students ( *n* = 133) on a five-point scale from strongly disagree to strongly agree.

However, the positive response to the induction course was not reflected in the Net Promoter Score, which was -8. This was calculated as the percentage (%) of ‘promoters’ (27.8%, 37/133 participants, who gave a score of 9 or 10) minus the percentage of ‘detractors’ (35.5%, 47/133 participants, who scored between 0-6) in response to the question ‘How likely is it that you would recommend this course to another student starting first year?’ The majority of survey participants (36.8%, 49/133) gave a score of 7 or 8 and thus were not included in the Net Promoter Score calculation.

**Figure 2:**
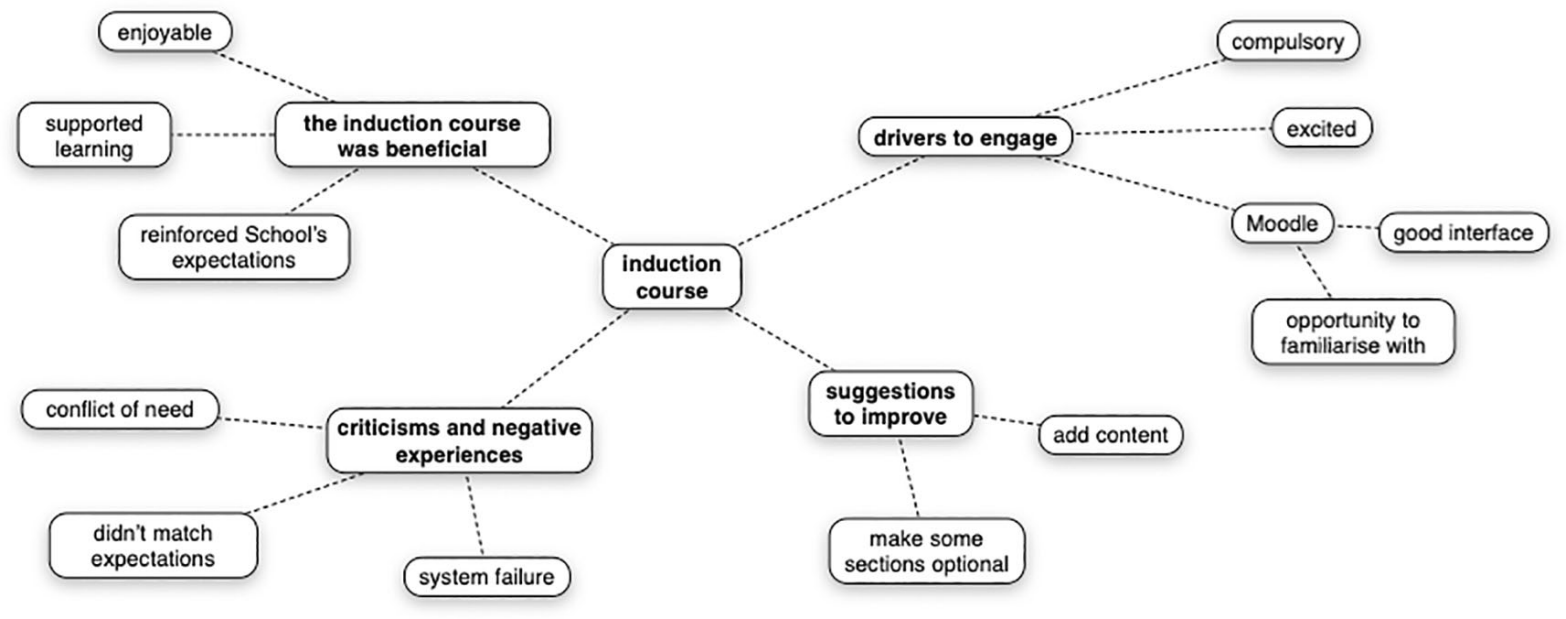
Summary of themes and codes that captured students’ perceptions of the induction course

Students’ experiences of accessing an online induction course, and the perceived utility of the course in preparing them for first year medicine was explored in depth during a focus group discussion. Four themes were identified that captured students’ perceptions of the course and to which all codes could be assigned ( Figure 2).

Four themes captured students’ perceptions ( *n* = 2) of the online induction course: ‘the induction course was beneficial’, ‘drivers to engage’, ‘criticisms and negative experiences’ and ‘suggestions to improve’. All codes assigned to data during the analysis process were assigned to a theme.

Focus group participants described their interaction with the induction course as an overall positive experience. Students highly valued the induction course in setting expectations both of themselves and the School, and providing an opportunity to orientate themselves with the digital skills, terminology and software necessary for them to succeed in their studies.

‘“this is what we expect of you”...it gave me quite a good first impression of how the medical school would be organised.’

Students were initially motivated to engage with the induction course because they were excited to begin medical school but were also cognizant that engagement with the induction course was expected of them.

‘I was like, “I’m not gonna, like start off on the wrong foot you know, you’ve worked so hard to get here.”’

Furthermore, students valued the opportunity to familiarise themselves with the virtual learning environment (Moodle) that they would be using throughout their University career. These factors likely influenced students’ on-going engagement with the course.

‘It was...easy to like navigate and then that did kind of like set you up to navigate Moodle later on.’

Students’ initial expectations of the course were mixed; one participant commented that the course was different from what they were expecting and that they thought the course was going to be *‘a bit more medical’* and focused on content of their degree programme, rather than foundational digital skills and a course overview. Situating the induction course in the context of digital skills awareness and foundational training will be important going forward to address this disconnect. Some minor system failures, e.g. content not loading, were also identified by a small number of students. However, a major challenge for the course creators echoed by the focus group participants was providing enough foundational information to support students with limited digital skills whilst not being too basic for others (the ‘conflict of need’). Participants recommended addressing this by making some sections within the course optional to complete.

‘I guess I was just a bit surprised that there was so much information that many students might not have known, but I understand that the point of that induction is to get everyone up to the same speed.’

Suggestions for course improvements were made both in the anonymous survey and during the focus group discussion, the themes of which overlapped. Students highlighted that inclusion of current students’ testimonies and ‘top tips’ would improve the course. A need for practical guidance such as an equipment list, and an overview of key terminology that they are likely to encounter early in their studies was also highlighted. Furthermore, focus group participants suggested better integration and signposting to the induction course during face-to-face workshops throughout the academic year to prompt students to return to the course to supplement their own learning.

## Discussion

There was a high level of engagement with the online induction course by first year undergraduate medicine (MBChB) students. Feedback from students indicates that the induction course supports academic preparation by setting realistic expectations and promoting academic and digital skills development. The current study adopted two approaches to data collection: firstly, a questionnaire drew responses from 42% of the year group and highlighted aspects of the course that the students found most helpful. Secondly, a focus group provided rich data regarding student perceptions of the online induction course and its utility throughout the first year of study.

The results of the online survey indicated that students’ training background influenced their perceived preparedness on entering the undergraduate medical course ( Table 2). The greatest proportion of students who had completed a foundation, pre-med or undergraduate course felt ‘very’ prepared on entering medical school (foundation or pre-med: 50.0%, 7/14; graduates: 54.2%, 13/24). Students who were entering medical education directly from school most often felt ‘moderately’ prepared (47.1%, 41/87). The majority of survey respondents were school leavers, which is representative of undergraduate medicine cohorts ( Moro and McLean, 2017). Further work might usefully explore these relationships using inferential statistics.

Course content on professionalism was most highly valued by students (90.9% students felt that the course gave them a sense of the professional expectations of them). Our graduating doctors are expected to demonstrate professional values and behaviours ( General Medical Council, 2020); embedding a culture of professionalism at this early stage of medical students’ careers highlights its significance.

Conversely, the survey responses indicated that participants felt that the induction course did not contain all the information that students needed (only 44% of survey participants felt that it did). This finding was in line with the negative Net Promoter Score (-8) given by the students, which may be explained, at least in part, by the theme ‘conflict of need’ highlighted in the focus group discussion. Offering students choice in how they engage with the induction course, by making some sections optional, will hopefully address this.

In the study focus group and survey results, students highlighted a desire for the induction course to include current student perspectives and guidance in future versions of the course. Indeed, peer-led support has been effective during induction in other professional degree programmes ( Paterson *et al.*, 2019). Other suggested materials that could easily be included in future versions of the course included an equipment list and an introduction to key terminology. These resources may be particularly pertinent for students first in their family to attend university who therefore cannot receive guidance from family members ( Brosnan *et al.*, 2016). Similarly, participants valued the opportunity to familiarise themselves with the Moodle site before commencing the course. In this way, the online induction course helped students formulate their student identity and supported their academic and digital skills development, which are key components of student transition into higher education ( Gale and Parker, 2012; Thomas, 2012).

A limitation of the focus group was the small number of student participants ( *n* = 2) who reported overall positive experiences of the induction course. Unfortunately, any opportunity to host additional focus groups was lost when face-to-face teaching stopped at our institution in mid-March due to COVID-19. The experiences of students who, for example, did not feel that the online induction course assisted their transition into medical school (responded ‘disagree’ or ‘strongly disagree’: 9.0%, 12/133) could be usefully explored in further research. It is reassuring however to note that the key themes identified in the focus group overlapped with many comments submitted to the online questionnaire. Analysis of the evidence from the ‘What Works? Student Retention and Success’ projects ( Thomas, 2012) suggests that the most effective pre-entry interventions combine the following roles: a) providing information; b) informing expectations; c) developing academic skills; d) building social capital (links with peers, current students and staff) and e) nurturing a sense of belonging. Findings from the current study indicate that students valued the online induction course because it aligned with these needs.

Whilst it is always imperative that online courses are revised in response to student feedback and to maintain up-to-date materials, it is now likely that remote and online delivery will be the primary mode of induction for incoming higher education students in September 2020. Additional systems of support will be needed to build a sense of community in the online space for students will be joining the University virtually. Continuing to collate resources and share best practice will be key to our collective success in this pivot to online delivery.

## Conclusion

The online induction course benefited students by helping them understand institutional expectations and providing the opportunity to identify their digital skills needs for them to succeed in their studies. The findings of this study indicate a need to customise support available to students to ensure that an appropriate level of information and support is offered. As we prepare for remote and/or blended delivery of our course content for academic session 2020-21, supporting student transition to online learning, scaffolding development of digital skills and fostering a sense of community is essential.

## Take Home Messages

• An online induction course is a feasible and acceptable method to support students during transition into undergraduate medical school.
• Future iterations of the course should include input from current students, such as video testimonies and perspectives.
• Online formats are likely to the primary format by which we induct our students for the upcoming academic year in response to the COVID-19 pandemic.

## Notes On Contributors

**Dr Kirsty McIntyre**, BSc (Hons), PhD, FHEA is a lecturer in the College of Medicine, Veterinary and Life Sciences, University of Glasgow. ORCiD: https://orcid.org/0000-0003-3224-7043
