# Supplementary material for: Bridging the gap: implementation of an online induction course to support students’ transition into first year medicine
Source: MedEdPublish (2016). 2021 Mar 17;9:193. Originally published 2020 Sep 14. [Version 2] doi: 10.15694/mep.2020.000193.2 (PMC10699373; doi:10.15694/mep.2020.000193.2)
Supplement: Peer review history

# Reviewer report: Reviewer response for version 2

**DOI:** 10.21956/mep.20277.r31479

**Published:** 2021-03-23

**Authors:** Megan Anakin

**Affiliations:** 1 University of Otago

This review has been migrated. The reviewer awarded 4 stars out of 5 Thank you for publishing this revised version of your article. I appreciate the time you spent addressing the comments and suggestions from reviewers. This article has now been strengthened and presents the data you collected and analysed in way that provides insights into the challenges of providing induction materials to students. The online context of this article and lessons learned are even more relevant to readers given the rapid shift educators have made to online learning globally over the past year. I will be sharing this article with my colleagues and discussing how we can better enhance our students’ transition our medical programme.I look forward to reading about the next steps of your research in this area.

Reviewer Expertise:

NA

No decision status is available

# Reviewer report: Reviewer response for version 2

**DOI:** 10.21956/mep.20277.r31478

**Published:** 2021-03-18

**Authors:** Barbara Jennings

**Affiliations:** 1 Norwich Medical School

This review has been migrated. The reviewer awarded 4 stars out of 5 As with version 1 of the article, https://doi.org/10.15694/mep.2020.000193.1, I think this is a very helpful and well written paper describing an online induction course and its evaluation. It will be of particular interest & use to curriculum leads for admissions and leads of early undergraduate years of study.The author has responded thoroughly to the reviewer comments and documented all amendments that were feasible between the article versions. The clarifications about governance steps and the format changes are also helpful – I can see the benefits of the structured abstract suggested by one of the v1 reviewers. Thank you to the author for this nice paper and its development.

Reviewer Expertise:

NA

No decision status is available

# Reviewer report: Reviewer response for version 1

**DOI:** 10.21956/mep.19009.r27276

**Published:** 2020-09-18

**Authors:** Megan Anakin

**Affiliations:** 1 University of Otago

This review has been migrated. The reviewer awarded 3 stars out of 5 Thank you for inviting me to review your research article. At my medical school, our reading group is discussing transition. Overall, this article is informative, however, several improvements are suggested before I feel comfortable sharing this article with my colleagues.Please consider adding subheadings in your abstract to help the reader appreciate the main components of your article at a glance. Please also consider removing the examples because these are details that require more explanation than can be accommodated by an abstract. Instead, consider include a statement about the sample and data analysis methods used.In the second sentence of the introduction, please support the absolute statement with a reference to substantiate the claim about all incoming undergraduates. At the end of the second paragraph, please consider explaining relevant details of the many studies that have investigated transition so that the reader is introduced to main factors and how they might be examined in your study. To help the reader better understand the context of your study, please consider describing the content of components of the five-unit online induction course and explaining how they relate to the transition issues identified in the literature and experienced by your students. Please consider ending the introduction section by stating the three aims of your study and then move the three final sentences of the last paragraph in that section into the methods section because they are about the methods.Please describe the methods used to analyse the quantitative and qualitative survey data in more detail help the reader understand why the decision was made to only provide descriptive statistics and not any inferential statistical tests to make comparisons among the sub-groups that are reported in the results section. In the results section, please report demographic information about your participants such as gender, ethnicity, previous education or entry pathway into the programme, so the reader is familiar with the features of the students sampled and the subgroups that are used to report the results. Please consider revising the statement about responses being ‘overwhelmingly positive’ because approximately one-third of responses were not agree or strongly agree. Given the statement in the introduction about 1-14% of medical students in the UK leaving before graduation, it is concerning that, on average, the results in Table 1 show approximately 10% of responses were disagree or strongly disagree and these results are not followed up in the discussion.In the discussion, please consider revising the statements in the second paragraph because no statistical comparisons were made among the subgroups of students to support the comparative claims about the results. An important limitation of this study to discuss is that the two students in the focus group found the course an overall positive experience and not students with negative experiences.Please consider revising the take home messages so that they more specifically reflect the evidence presented in this research article.I would be very happy to read and review a revised version of this article.

Reviewer Expertise:

NA

No decision status is available

# Reviewer report: Reviewer response for version 1

**DOI:** 10.21956/mep.19009.r27275

**Published:** 2020-09-18

**Authors:** Barbara Jennings

**Affiliations:** 1 Norwich Medical School

This review has been migrated. The reviewer awarded 4 stars out of 5 This was a very useful paper to read. The literature that was cited about transitions into higher education, and the concepts explained in the introduction, will be very valuable for academic teams. Particularly to those interested in induction and signposting at a time when effective remote teaching and learning methods are in demand.I will certainly be recommending it to our admissions and year-one academic teams for discussion. The main aim of the research was to evaluate perceptions about the benefits of an online induction course. The methodology was clearly described and the supplementary file was helpful. The authors also describe exemplary research governance steps and present the main results clearly in table and figure 1. Interesting findings were also illustrated from the thematic analysis presented in figure 2. Some conflicts between expectations and experiences of learners were explored in the discussion as was a limitation to the study- which was the small uptake of participants in the focus group (any opportunity for recruitment to additional focus groups being another covid-casualty).The conclusions from the authors about the acceptability of the induction course are backed up by the survey responses and uptake of course materials. The introduction also reminded me of an important message for the coming academic year, which is not all teaching materials have to be created from scratch, there is a wealth of resources that can be shared and reused (these authors created “a new five-unit online induction course using existing resources available under creative commons licensing”). Well done for the course development, and for the well-organised evaluation described in this article.

Reviewer Expertise:

NA

No decision status is available
